# Retention of Teeth after Necrosis of Jaw

**Published:** 1859-04

**Authors:** 


*Retention of Teeth after Necrosis of Jaw.*—Some interesting cases of this sort, have been published in the Medical Times and Gazette, appropos of one that occurred in the practice of Mr. Skey, at St. Bartholomew's. The patient had necrosis of a large portion of the lower jaw. The sequestrum, which was loose, and consisted of the whole left side from the ramus to the symphysis and of the right, as far as the first molar tooth, was easily removed. Upon examination this sequestrum was found to contain the sockets for twelve teeth, viz. for the whole of those on the left side, and for the incisors, canine and first bicuspid of the right. On the right side a part of the alveolar border appeared to have escaped the necrosis. Four months after the removal of the dead bone, the man again presented himself, asking to have his teeth removed. Strange to say, the incisors, canine and bicuspid of the right side remained firmly attached to the gum and evidently alive. The gums had sunk and become distorted, and the teeth having no bony support, and standing very irregularly, were only in the patient's way. Four of them were removed, the fifth (the right first bicuspid) being allowed to remain, because it was firmer than the others. The patient is described as a delicate, unhealthy young man, of about twenty years of age. The periosteum remained.

